# Exploring the risk of infection events in patients with asthma receiving *anti*-IL-5 monoclonal antibodies: A rapid systematic review and a meta-analysis

**DOI:** 10.1016/j.heliyon.2023.e23725

**Published:** 2023-12-15

**Authors:** Riccardo Giossi, Arianna Pani, Jan Schroeder, Francesco Scaglione

**Affiliations:** a Chemical-Clinical Analyses Unit, ASST Grande Ospedale Metropolitano Niguarda, Milan, Italy; b Department of Oncology and Hemato-Oncology, Università Degli Studi di Milano, Milan, Italy; c Allergology and Immunology Unit, ASST Grande Ospedale Metropolitano Niguarda, Milan, Italy

**Keywords:** Benralizumab, Mepolizumab, Reslizumab, IL-5, Infection, Meta-analysis

## Abstract

**Introduction:**

Benralizumab, mepolizumab, and reslizumab are novel monoclonal antibodies approved for asthma, targeting eosinophilic inflammation. Benralizumab is directed against IL-5 receptor (IL-5R), while mepolizumab and reslizumab are directed against IL-5. The three drugs cause a reduction in eosinophils, but benralizumab also causes a cytotoxic effect on eosinophils and basophils. Recently, it has been reported that suboptimal responders to benralizumab presented exacerbations associated with concomitant infections and sputum neutrophilia and the incidence of infections was greater in patients receiving benralizumab compared to mepolizumab and reslizumab. For this reason, we wanted to explore potential differences in terms of infectious adverse events between the three different *anti*-IL-5 antibodies.

**Methods:**

We performed a rapid systematic review on PubMed up to April 28, 2022. We included randomized controlled trials (RCTs) evaluating benralizumab, mepolizumab, or reslizumab in patients with asthma. Included outcomes were the reporting of any respiratory tract infection and any emergency department (ED) or hospital admission for infection or asthma exacerbation. A Mantel-Haenszel meta-analysis was performed with Cochrane RevMan 5.4 to estimate pooled odds ratios (OR) with 95 % confidence intervals (CI). A subgroup analysis for the different active treatments was performed.

**Results:**

From 163 references we included 21 studies reporting the results of 23 different RCTs for a total population of 9156 patients. All studies compared *anti*-IL-5 antibodies against placebo. *Anti*-IL-5 treatment resulted in non-significant differences compared to placebo in the odds for nasopharyngitis (OR = 0.90; 95 % CI from 0.76 to 1.07), pharyngitis (OR = 1.45; 95 % CI from 0.92 to 2.28), upper respiratory tract infection (URTI) (OR = 0.97; 95 % CI from 0.82 to 1.15), rhinitis (OR = 1.01; 95 % CI from 0.71 to 1.44), pneumonia (OR = 0.56; 95 % CI from 0.10 to 2.01), and influenza (OR = 0.84; 95 % CI from 0.65 to 1.09). We observed significant reductions in the reporting of sinusitis (OR = 0.75; 95 % CI from 0.53 to 1.06), bronchitis (OR = 0.71; 95 % CI from 0.59 to 0.86), and ED or hospital admission due to asthma exacerbation for overall *anti*-IL-5 antibodies compared to placebo (OR = 0.59; 95 % CI from 0.40 to 0.88). We were not able to discriminate whether exacerbations were associated with infections or to increased sputum eosinophilia. From the subgroup analysis, we observed differences in directions and magnitudes of the effect size in the reporting of some events. Benralizumab was associated with increased odds of pharyngitis (OR = 1.56; 95 % CI from 0.97 to 2.52) and a similar trend was observed for mepolizumab in the reporting of rhinitis (OR = 1.85; 95 % CI from 0.72 to 4.78), both non-statistically significant. In terms of effect size, benralizumab also showed higher odds for bronchitis and pneumonia in comparison to mepolizumab and reslizumab (OR = 0.76, OR = 0.69, and OR = 0.60 for bronchitis and OR = 0.80, OR = 0.20, and OR = 0.45, respectively, all non-significant).

**Conclusion:**

*Anti*-IL-5 treatments might have different effects on the reporting of some infection events in patients with asthma. However, the evidence is limited by sample size and far than conclusive and suggest the need of future studies to evaluate the risk of infections in patients with asthma receiving *anti*-IL-5 treatments.

## 1 Introduction

Novel treatments for asthma are directed against eosinophilic inflammation by targeting IL-5. IL-5 is a homodimeric cytokine which acts as the primary modulator of eosinophils. In Europe, three monoclonal antibodies directed against IL-5 have been approved. Mepolizumab and reslizumab bind directly to circulating IL-5 and reduce eosinophil counts by inhibiting IL-5 signalling. Benralizumab, on the other hand, is a monoclonal antibody directed against the IL-5 receptor (IL-5R) alpha chain with a cytotoxic effect capable of completely depleting the eosinophilic as well as basophilic population [1].

The role of eosinophils in relation to the pathophysiology of asthma has been extensively discussed in the literature [2]. A high eosinophil count is associated with more disease severity [3]. Eosinophils accumulate in the lungs in the inflammatory setting causing tissue damage and promoting Th2-mediated inflammatory signalling [4].

On the other hand, the role of eosinophils in protecting the body from infection remains debated. Traditionally, the function of eosinophils has traditionally been associated with protection against parasitic infections. However, studies have also shown they are involved in fungal [5,6] and viral infections [7]. For example, it has recently been reported that eosinopenia is linked with acute respiratory deterioration during SARS-CoV2 infection [8,9]. Other studies associated eosinopenia with poor outcomes in patients with acute exacerbations of chronic obstructive pulmonary disease (COPD) [10] and identified eosinopenia as a possible marker of severe infection and sepsis [11].

A recent prospective cohort study reported a suboptimal response, namely the presence of exacerbations or failure to reduce prednisone by at least 50 %, in about 27 % of patients receiving benralizumab. Authors found that only a minority of exacerbations in this group of patients were associated with sputum eosinophilia. Conversely, the majority of them were associated with concomitant infections and sputum neutrophilia. Also, the incidence of respiratory infections increased in the assessed population while receiving benralizumab and was significantly greater compared to that observed in a group of patients receiving mepolizumab or reslizumab [12].

Given these observations, we decided to conduct a rapid systematic review and a meta-analysis including randomized controlled trials (RCTs) evaluating *anti*-IL-5 monoclonal antibodies for asthma to explore potential differences in terms of the reporting of infectious adverse events.

## 2 Methods

### 2.1 Search strategy and selection criteria

We performed a literature search on PubMed from the beginning up to August 28, 2023 to include RCTs fulfilling our inclusion criteria. The search strategy included “benralizumab”, “mepolizumab”, “reslizumab”, “asthma”, and “RCTs” as keywords used as MeSH and free terms and combined with Boolean operators (Supplementary Material S1). We included full-text English articles reporting data of RCTs, in patients with asthma of any age, evaluating benralizumab, mepolizumab, or reslizumab against placebo or any active comparator. Observational studies, case series, case reports, reviews, editorials, commentaries, congress abstracts, and study with no outcomes were excluded. Two study authors (R.G. and A.P.) independently screened literature citations for inclusion and discrepancies were resolved by collegial discussion.

### 2.2 Assessed outcomes

Included outcomes of our systematic review were the reporting of any respiratory tract infection coded according to the MedDRA dictionary. Infection events were collected separately for each available category from included RCTs. Additional outcomes were emergency department (ED) or hospital admission for infection and ED or hospital admission asthma exacerbation. Respiratory tract infections were defined as upper respiratory tract infections (URTI), including nasopharyngitis, pharyngitis, sinusitis, acute sinusitis, and rhinitis and lower respiratory tract infections (LRTI), such as bronchitis, pneumonia, and influenza.

### 2.3 Data extraction

Two study authors (R.G. and A.P.) independently extracted included study data on an Excel spreadsheet and discrepancies were resolved by discussion. Extracted data consisted in: first author, year, period, study duration, patients age and sex, RCTs main inclusion and exclusion criteria, total patient included, the different treatment and *anti*-IL-5 dose regimens, and prespecified included outcomes.

### 2.4 Statistical analysis

Events from outcomes of interest were collected from included studies. Whenever the number of events was not completely reported, we estimated the number of adverse events from available rates or odds ratios (ORs), confidence intervals (CI), or p-values. For all comparisons, we used a random-effect Mantel-Haenszel meta-analysis performed with Cochrane RevMan 5.4 software. Heterogeneity was evaluated with the I-squared statistic. Pooled estimates were reported as ORs and 95%CI. A subgroup analysis was performed based on the different *anti*-IL-5 to allow comparisons in-between treatments.

### 2.5 Role of the funding source and ethical considerations

The study was not sponsored and did not receive any funding. Due to the nature of the study on already published data without the involvement of new human participants or animals an IRB approval was not necessary.

## 3 Results

### 3.1 Search results and study characteristics

**Fig. 1:**
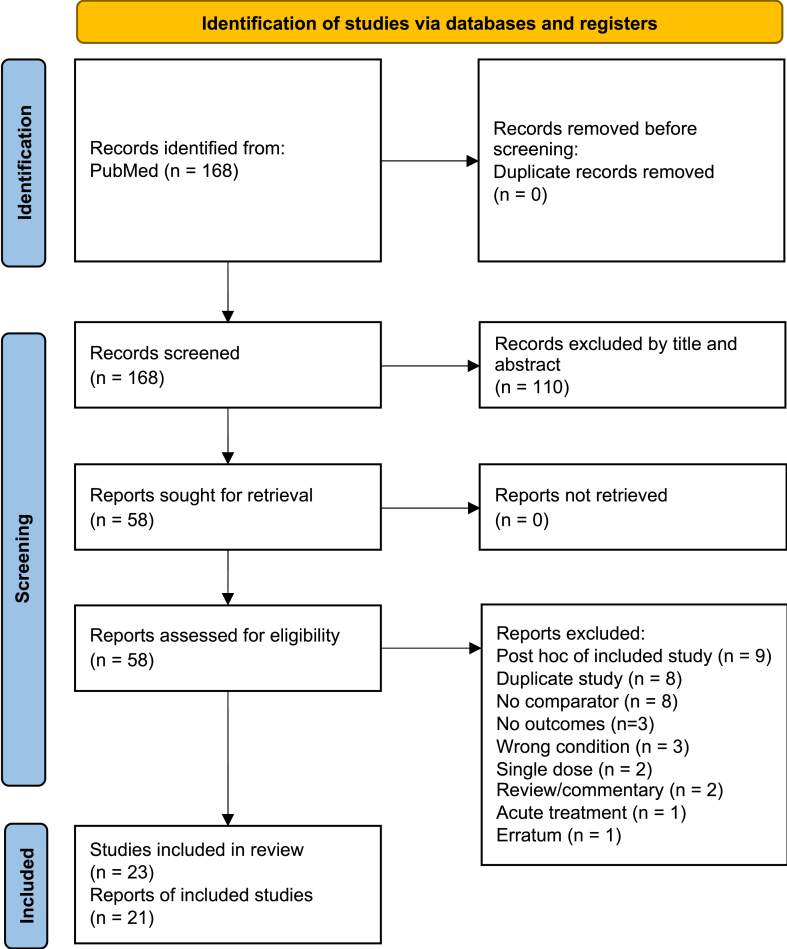
PRISMA flow diagram.

From a total of 168 retrieved references, 110 were excluded by title and abstract and 58 were read in full text. Of these, 37 were excluded with reasons and a final number of 21 studies reporting the results of 23 different RCTs were included (Fig. 1). Of these, 11 RCTs were on benralizumab [[13], [14], [15], [16], [17], [18], [19], [20], [21], [22]], 6 on mepolizumab [[23], [24], [25], [26], [27], [28]], and 6 on reslizumab [[29], [30], [31], [32], [33]], all compared to placebo. The total number of patients was 9156 across all included studies and 12 studies enrolled also paediatric patients from the age of 12 years. Benralizuamb was administered to 2992 patients; 1231 patients received mepolizumab, 1462 reslizumab, and 3471 a placebo. Included studies characteristics are described in Table 1.

**Table 1:** Included studies characteristics.

Author Year	Inclusion criteria	Study duration/phases	Treatment	Comparator	Total population	Female, N	Steroids and concurrent therapy management
***Benralizumab***							
Laviolette 2013 [13]	M and F, 18 to 65 yo, with eosinophilic asthma, on a stable asthma medication for 4 weeks before screening	86 to 56 + 86 days	Benralizumab 1 mg/kg ev once, or 100 or 200 mg in 4 sc injections on days 0, 28 and 56	Placebo	27	16	Usual concurrent therapy
Castro 2014 [14]	M and F, 18 to 75 yo, with EO or non-EO asthma, with 1 year treatment with ICS/LABA, and two to six exacerbations needing systemic corticosteroids in the last year	52 w	EO: Benralizumab 2 mg, or 20 mg, or 100 mg; non-EO: Benralizumab 100 mg. Two sc every 4 w for the first 3 doses, then every 8 weeks	EO and non-EO: Placebo	606	417	Stratification based on medium or high steroid dose
Park 2016 [15]	M and F, 20 to 75 yo, with EO asthma, on ICS/LABA combination, 2–6 exacerbations requiring systemic CS in the past year	52 w	Benralizumab 2, 20, or 100 mg sc q4w up to w8, then q8w	Placebo	103	65	Stratification based on medium or high steroid dose
FitzGerald 2016 (CALIMA) [16]	M and F, 12 to 75 yo, with asthma, on medium dose ICS/LABA, two or more exacerbations in the last year requiring systemic CS or temporary ioncrease of usual oral CS	56 w	benralizumab 30 mg q4w, 30 mg q4w for three doses and then q8w	Placebo	1306	807	Stratification based on medium or high steroid dose
Bleecker 2016 (SIROCCO) [17]	M and F, 12 to 75 yo, with asthma, on high dose ICS/LABA, two or more exacerbations in the last year requiring systemic CS or temporary increase of usual oral CS	48 w	Benralizumab 30 mg q4w, 30 mg q8w	Placebo	1204	796	Usual concurrent stable therapy
Ferguson 2017 (BISE) [18]	M and F, 18 to 75 yo, mild to moderate asthma, low to medium dose ICS or low dose ICS/LABA, night time or daytime asthma symptom score 1+ for 2+ days, or rescue SABA for 2 days, or night time awakenings due to asthma, in the last 7 days	20 w	Benralizumab 30 mg q4w	Placebo	211	129	Usual concurrent stable therapy
Nair 2017 (ZONDA) [19]	M and F, adult, with asthma and eosinophils more than 150/μL, on oral CS for 6 months and LABA	36 w	Benralizumab 30 mg q4w, 30 mg q8w	Placebo	220	135	Usual concurrent therapy with modifiable CS dose
Zeitlin 2018 (ALIZE) [20]	M and F, 12 to 21 yo, with asthma and current regular use of ICS, all receiving quadrivalent influenza vaccine in the trial context	20 w	Benralizumab 30 mg sc q4w; influenza quadrivalent vaccine	Placebo; influenza quadrivalent vaccine	103	42	ICS at stable dose
Panettieri 2020 (SOLANA) [21]	M and F, 18 to 75 yo, with severe eosinophilic asthma, on ICS/LABA, and two exacerbations requiring systemic CS or increase in maintenance oral CS in the last year	16 w	Benralizumab 30 mg q4w	Placebo	233	157	Usual concurrent therapy
Harrison 2021 (ANDHI) [22]	M and F, 18 to 75 yo, with severe eosinophilic asthma, on ICS and additional controllers, and two asthma exacerbations in the last year	24 w	Benralizumab 30 mg q8w	Placebo	660	399	Usual concurrent stable therapy
***Mepolizumab***							
Flood-Page 2003 [23]	M and F, 18 to 55 yo, with mild asthma and atopic by prick test to one or more aeroallergens, well controlled with SABA and no use of CS or other anti-inflammatory drugs in the previous 8 weeks	20 w	Mepolizumab 750 mg q4w ev	Placebo	24	7	NO
Haldar 2009 [24]	M and F, more than 18 yo, with refractory asthma, sputum eosinophil more than 3 % despite high dose steroids, at least 2 exacerbations requiring rescue prednisolone in previous 12 months	12 months	Mepolizumab 750 mg monthly	Placebo	61	29	Usual concurrent therapy
Pavord 2012 (DREAM) [25]	M and F, 12 to 74 yo, with refractory asthma, evidence of eosinophilic inflammation, two or more exacerbations requiring systemic CS in the previous year, treated with inhalatory steroids and requiring additional controller drugs.	52 w	Mepolizumab 75/250/750 mg monthly	Placebo	616	393	Stable treatment of at least 880 μg fluticasone propionate equivalent per day, with or without maintenance oral CS, and additional controller drugs
Ortega 2014 (MENSA) [26]	M and F, 12 to 82 yo, with eosinophilic asthma, two or more exacerbations requiring systemic CS in the previous year while on 880 μg fluticasone or equivalent and an additional controller	1-6 w run-in; 32 w treatment - 8 w safety	Mepolizumab 75 mg ev, or 100 mg sc every 4 w	Placebo	576	329	Usual concurrent therapy
Bel 2014 (SIRIUS) [27]	M and F, 12 yo or older, with eosinophilic severe asthma, six months maintenance treatment with inhaled and systemic steroids and an additional controller	Glucocorticoid optimization; Randomization and induction 4 w; glucocorticoid reduction 16 w; maintenance 4 w; final safety at week 32	Mepolizumab 100 mg sc	Placebo	135	74	Optimized dose
Chupp 2017 (MUSCA) [28]	M and F, 12 yo or older, with eosinophilic asthma, on high dose ICS plus other controllers, and at least two exacerbations requiring treatment in the last year	24 w	Mepolizumab 100 mg sc	Placebo	556	325	Usual concurrent therapy; only 25 % with maintenance OS
***Reslizumab***							
Castro 2011 [29]	M and F, 18 to 75 yo, with poorly controlled asthma, receiving high-dose ICS and at least one other agent	15 w	Reslizumab 3,0 mg/kg	Placebo	106	63	High-dose ICS (≥440 μg of fluticasone twice per day) in combination with at least one other agent
Castro 2015 (Study 1) [30]	M and F, 12 to 75 yo, with eosinophilic asthma, on ICS with or without another controller, and at least one exacerbation requiring systemic steroids in the last year	52 w treatment and last visit 90 days after EOT	Reslizumab 3,0 mg/kg q4w	Placebo	489	303	Usual concurrent therapy
Castro 2015 (Study 2) [30]	M and F, 12 to 75 yo, with eosinophilic asthma, on ICS with or without another controller, and at least one exacerbation requiring systemic steroids in the last year	52 w treatment and last visit 90 days after EOT	Reslizumab 3,0 mg/kg q4w	Placebo	464	294	Usual concurrent therapy
Bjermer 2016 [31]	M and F, 12 to 75 yo, with inadequately controlled eosinophilic asthma, on ICS	16 w + 4 w follow up	Reslizumab 0,3 mg/kg or 3,0 mg/kg q4w ev	Placebo	315	174	Usual concurrent therapy
Corren 2016 [32]	M and F, 18 to 65 yo, with inadequately controlled asthma (patients were not selected on the basis of eosinophyls), on ICS	16 w treatment + 12 w follopw up	Reslizumab 3.0 mg/kg ev q4w	Placebo	496	315	Usual concurrent therapy
Bernstein 2020 (Study 1) [33]	M and F, 12 yo or older, with eosinophilic asthma, on medium-dose ICS and at least one other controller, and two exacerbations requiring systemic CS in the last year	52 w	Reslizumab 110 mg q4w	Placebo	468	308	ICS, some OS
Bernstein 2020 (Study 2) [33]	M and F, 12 yo or older, with eosinophilic asthma, on medium-dose ICS and a daily maintenance OCS, at least 6 months high-dose ICS in the six months before screening at least one other controller, and two exacerbations requiring systemic CS in the last year	24 w	Reslizumab 110 mg q4w	Placebo	177	145	OS all concurrent; stratified based on steroid dose; with modifiable CS dose

Abbreviations: CS = corticosteroids; EO = eosinophilic; ICS = inhalatory corticosteroids; LABA = long-acting beta agonists; SABA = short-acting beta agonists.

### 3.2 Upper respiratory tract infections

**Fig. 2:**
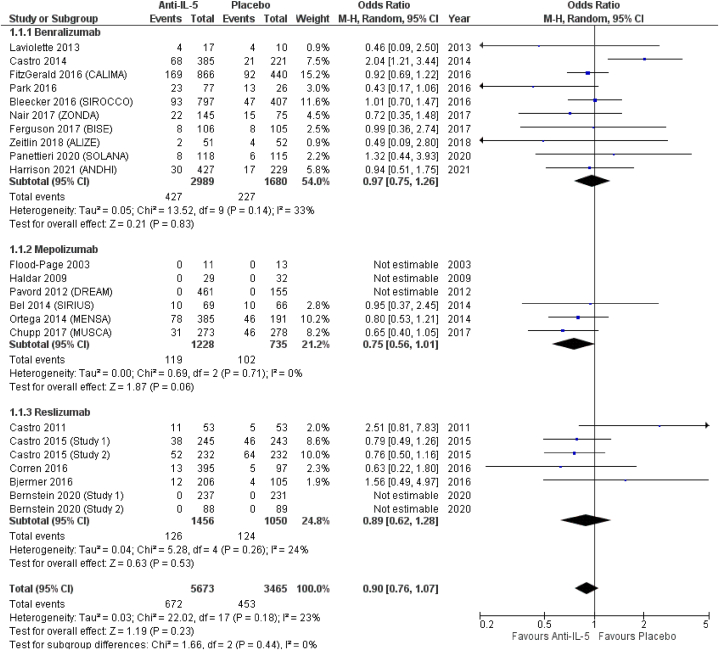
Nasopharyngitis.

**Fig. 3:**
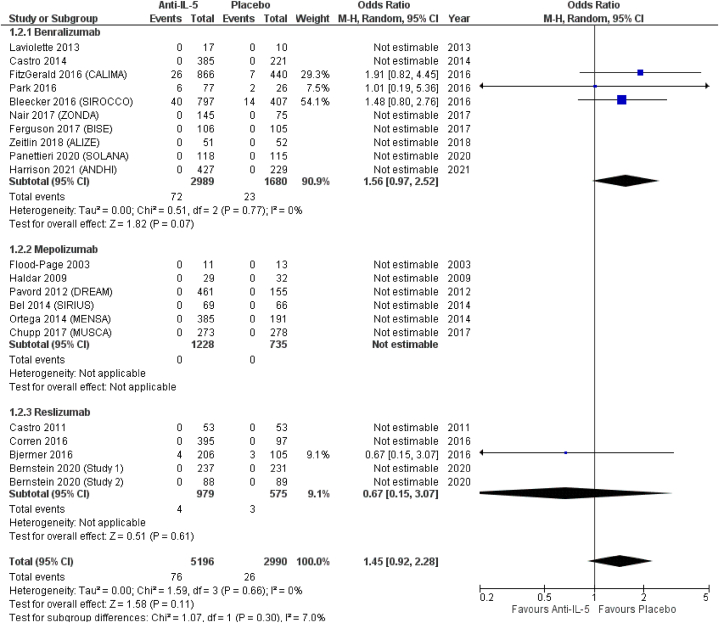
Pharyngitis.

**Fig. 4:**
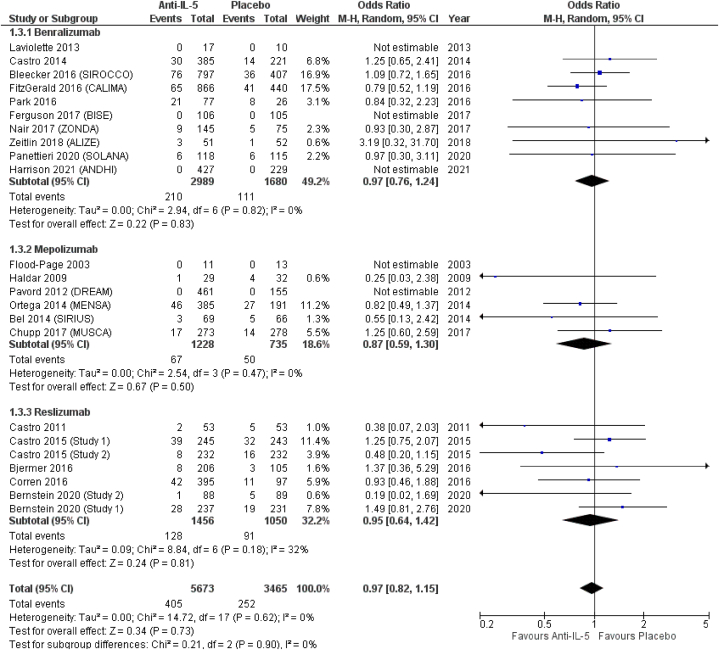
Upper respiratory tract infection.

**Fig. 5:**
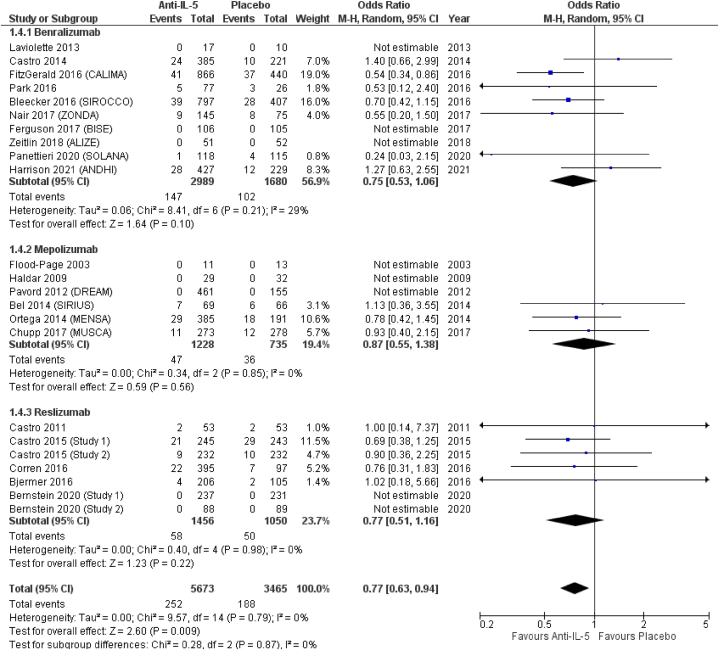
Sinusitis.

**Fig. 6:**
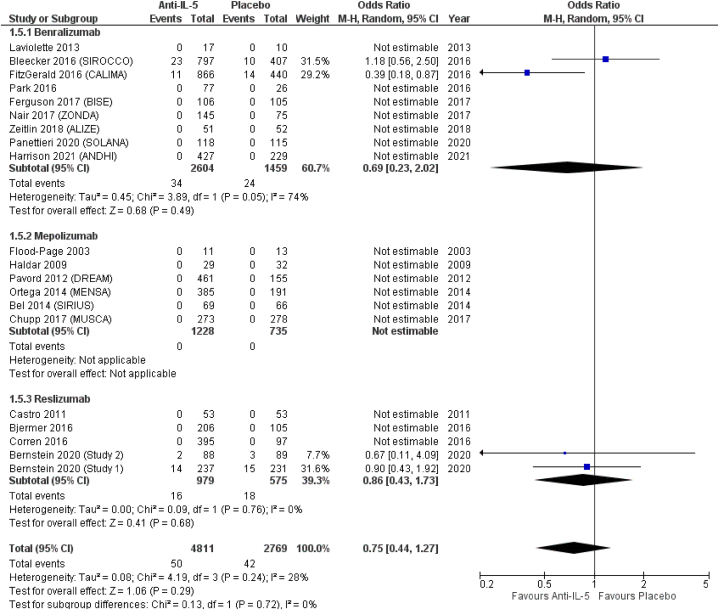
Acute sinusitis.

**Fig. 7:**
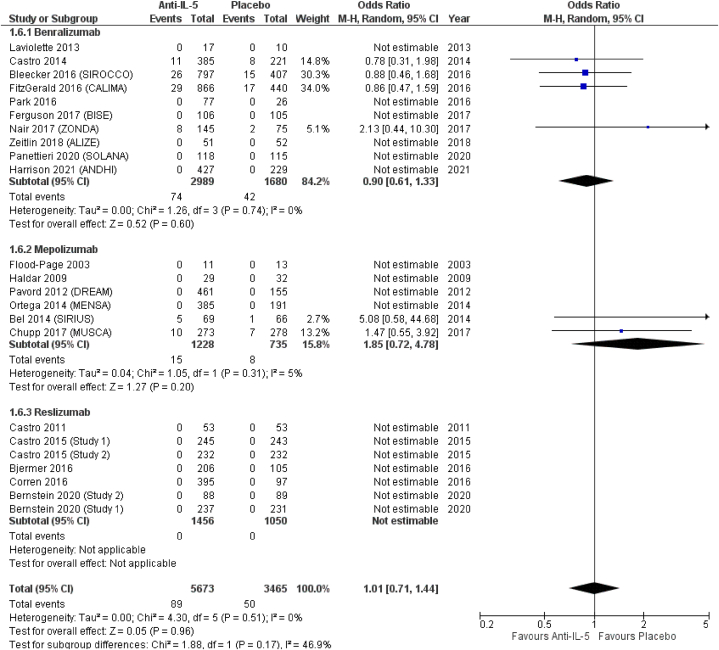
Rhinitis.

From included RCTs, we managed to extract data on the following upper respiratory tract infections: nasopharyngitis, pharyngitis, upper respiratory tract infection (URTI), sinusitis, acute sinusitis, and rhinitis. *Anti*-IL-5 treatment resulted in non-significantly reduced odds of nasopharyngitis (OR = 0.90; 95 % CI from 0.76 to 1.07), compared to placebo. In terms of effect size, among the three treatments, mepolizumab showed the lowest odds for nasopharyngitis (OR = 0.75; 95 % CI from 0.56 to 1.01), followed by reslizumab (OR = 0.89; 95 % CI from 0.62 to 1.28), and benralizumab (OR = 0.97; 95 % CI from 0.75 to 1.26), all non-significantly different compared to placebo (Fig. 2). *Anti*-IL-5 treatment resulted in non-significantly increased odds of pharyngitis (OR = 1.45; 95 % CI from 0.92 to 2.28). Pharyngitis was not reported by patients included in RCTs on mepolizumab (OR = not estimable), while benralizumab and reslizumab resulted in non-significantly increased (OR = 1.56; 95 % CI from 0.97 to 2.52) and decreased (OR = 0.67; 95 % CI from 0.15 to 3.07) odds for pharyngitis, respectively (Fig. 3). We found non-significant differences between *anti*-IL-5 treatment and placebo for the reporting of URTI (OR = 0.97; 95 % CI from 0.82 to 1.15). For the same outcome, we observed similar results in the subgroup analysis for benralizumab (OR = 0.97; 95 % CI from 0.76 to 1.24), mepolizumab.

(OR = 0.87; 95 % CI from 0.59 to 1.30), and reslizumab (OR = 0.95; 95 % CI from 0.64 to 1.42) (Fig. 4). *Anti*-IL-5 treatment significantly reduced the odds for sinusitis (OR = 0.77; 95 % CI from 0.63 to 0.94), compared to placebo. In terms of effect size, albeit not reaching statistical significance, benralizumab showed the highest odds for sinusitis (OR = 0.75; 95 % CI from 0.53 to 1.06), followed by reslizumab (OR = 0.77; 95 % CI from 0.63 to 0.94) and mepolizumab (OR = 0.87; 95 % CI from 0.55 to 1.38) (Fig. 5). Similar results were observed for acute sinusitis (Fig. 6). *Anti*-IL-5 treatment resulted in no significant difference from placebo for the reporting of rhinitis (OR = 1.01; 95 % CI from 0.71 to 1.44). For the same outcome, benralizumab resulted in non-significant reduction of rhinitis reporting (OR = 0.90; 95 % CI from 0.61 to 1.33), mepolizumab in non-significant increased (OR = 1.85; 95 % CI from 0.72 to 4.78) odds, while no rhinitis events were reported for reslizumab (OR = not estimable) (Fig. 7).

### 3.3 Lower respiratory tract infections and influenza

**Fig. 8:**
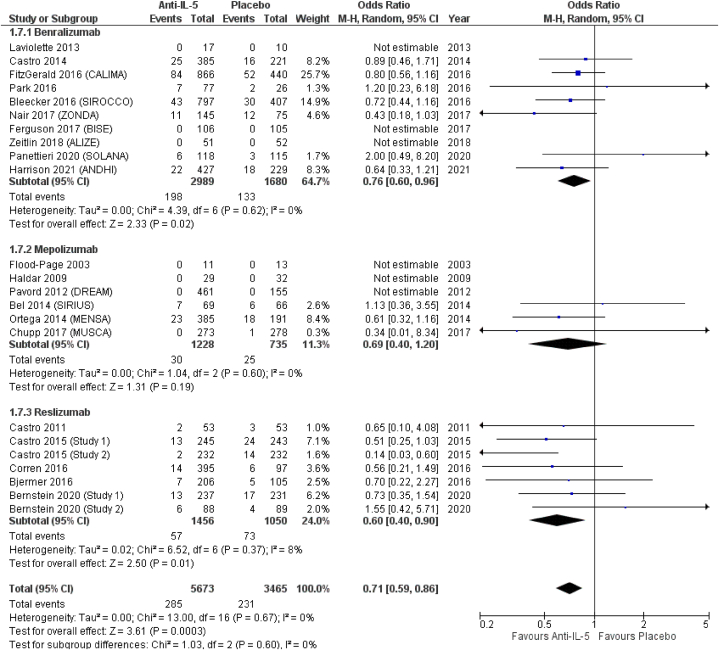
Bronchitis.

**Fig. 9:**
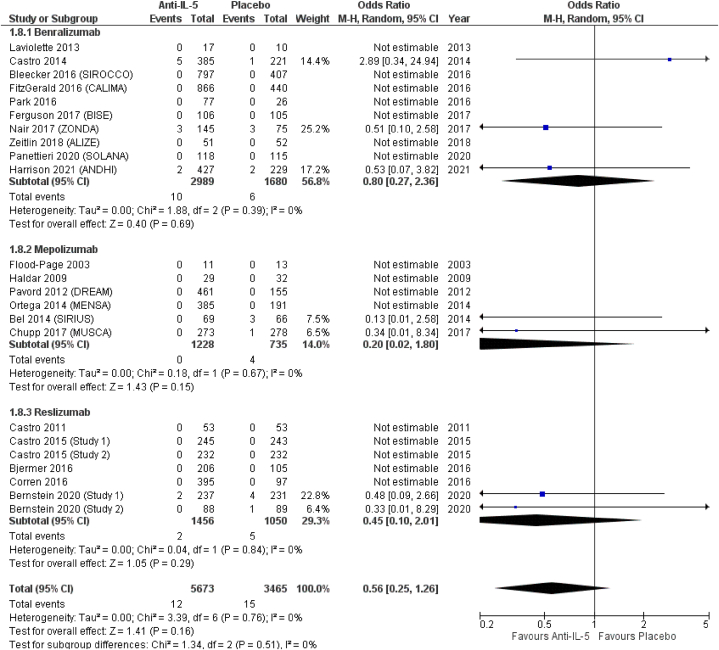
Pneumonia.

**Fig. 10:**
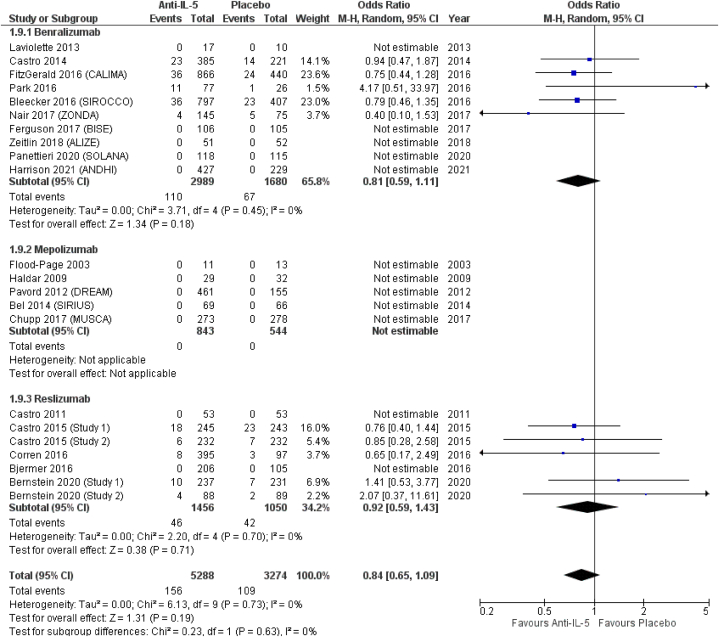
Influenza.

From included RCTs, we managed to extract data on the following lower respiratory tract infections: bronchitis, pneumonia, and influenza. *Anti*-IL-5 treatment was associated with a significant reduction of bronchitis (OR = 0.71; 95 % CI from 0.59 to 0.86). Similar results were obtained in the subgroup analysis, with, in order of effect size from the lower to higher, a significant reduction in the odds for benralizumab (OR = 0.76; 95 % CI from 0.60 to 0.96), a non-significant reduction in the odds of mepolizumab (OR = 0.69; 95 % CI from 0.40 to 1.20), and a significant reduction in the odds of reslizumab (OR = 0.60; 95 % CI from 0.40 to 0.90), compared to placebo (Fig. 8). Conversely, *anti*-IL-5 treatment resulted in non-significant reduction of pneumonia (OR = 0.56; 95 % CI from 0.10 to 2.01). In terms of effect size, among the three treatments, mepolizumab showed the lowest odds for pneumonia (OR = 0.20; 95 % CI from 0.02 to 1.80), followed by reslizumab (OR = 0.45; 95 % CI from 0.10 to 2.01), and benralizumab (OR = 0.80; 95 % CI from 0.27 to 2.36), all non-significantly different from placebo (Fig. 9). *Anti*-IL-5 treatment resulted in non-significantly reduced odds for influenza (OR = 0.84; 95 % CI from 0.65 to 1.09). Influenza was not reported by patients included in RCTs on mepolizumab (OR = not estimable), while benralizumab (OR = 0.81; 95 % CI from 0.59 to 1.11) and reslizumab (OR = 0.92; 95 % CI from 0.59 to 1.43) resulted in non-significantly reduced odds for influenza, respectively (Fig. 10).

### 3.4 ED or hospital admission

**Fig. 11:**
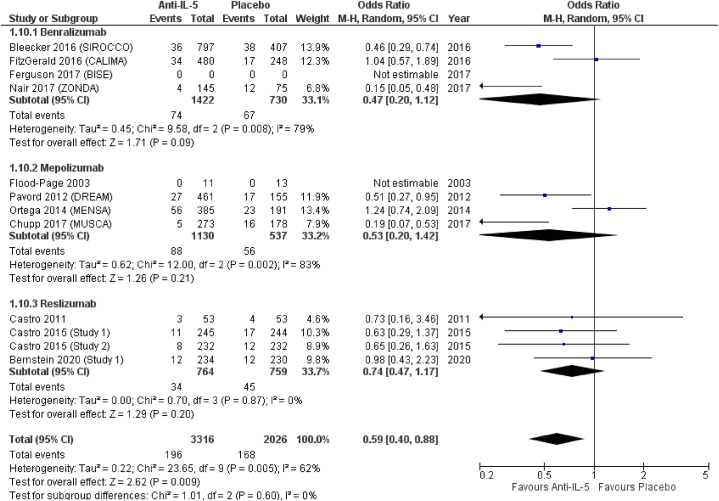
ED or hospital admission for asthma exacerbation.

In included studies, ED or hospital admission was reported as an outcome related to asthma exacerbation. On the contrary, ED or hospital admission for infection was not reported. Neither was it possible to extract data on whether an infection was present at the time of ED or hospital admission due to asthma exacerbation. This outcome was reported by 12 included RCTs [[16], [17], [18], [19],^23^,^25^,^26^,[28], [29], [30],^33^]. *Anti*-IL-5 antibodies were associated to reduced odds of ED or hospital admission for asthma exacerbation (OR = 0.59; 95 % CI from 0.40 to 0.88). In terms of effect sizes, benralizumab showed the lowest odds for ED or hospital admission due to asthma exacerbation (OR = 0.47; 95 % CI from 0.20 to 1.12), followed by mepolizumab (OR = 0.53; 95 % CI from 0.20 to 1.42) and reslizumab (OR = 0.74; 95 % CI from 0.47 to 1.17), all statistically non-significant (Fig. 11).

## 4 Discussion

We conducted a systematic review and a meta-analysis to explore the association of *anti*-IL-5 antibodies administration with the reporting of infective events in patients with asthma. From our analysis emerged a significant reduction in the reporting of sinusitis and bronchitis and a reduction in the odds for ED or hospital admission due to asthma exacerbation for overall *anti*-IL-5 antibodies compared to placebo. These observations are likely correlated to the mechanism of action of *anti*-IL-5 antibodies and reflect their clinical efficacy on eosinophilic inflammation. Indeed, *anti*-IL-5s are currently being evaluated also for chronic rhinosinusitis and nasal polyposis [[34], [35], [36]]. These conditions share physio-pathological mechanisms with asthma and are frequently reported in patients with asthma. Thus, the significant reduction of sinusitis observed in our study with *anti*-IL-5 antibodies may be related to the concurrent reduced inflammation of the nasal mucosa while the reduction of bronchitis likely reflects an improvement in the bronchial system's status.

The reporting of other included infection events (nasopharyngitis, pharyngitis, URTI, acute sinusitis, rhinitis, pneumonia, and influenza) was non-significantly different. However, we observed differences both in directions and magnitudes of some effect sizes. In particular, benralizumab was associated with increased odds of pharyngitis, although at limits of statistical non-significance; a similar trend was observed also for mepolizumab on the reporting of rhinitis. Similar to bronchitis, *anti*-IL-5 treatment was associated with a non-significant reduction in the odds for pneumonia. However, for both infections and more importantly pneumonia the ORs of benralizumab were closer to the line of non-significance compared to those of reslizumab and mepolizumab in terms of effect size, (i.e., benralizumab reduced less the occurrence of bronchitis and especially pneumonia). This trend, albeit limited by the sample size and the low number of reported patients with the event, was similar to that observed in the aforementioned prospective cohort study [12].

*Anti*-IL-5 treatment was associated with a significant reduction of ED or hospital admission for asthma exacerbation. In terms of effect sizes, the greatest reduction in ED or hospital admission was observed with benralizumab, followed by mepolizumab and reslizumab. This reduction was non-significant in all the three drug subgroups; however, this could be explained by the relatively low frequency of the observed outcome, leading to large confidence intervals. Of note, the cumulative effect sizes and their directions were consistent across the three cumulative subgroups. In this context, the comparison of the trend of the clinical efficacy (i.e., the reduction of ED or hospital admission for exacerbation) and the trend of the reporting of some infection events is conflicting. In terms of effect size and direction, on one side, drugs such as benralizumab and mepolizumab reduced exacerbations and *anti*-IL-5 treatment reduced sinusitis and bronchitis; on the other hand, benralizumab was less effective in reducing pneumonia and even seemed to increase pharyngitis. Also, we were not able to evaluate ED or hospital admission for infections and neither to discriminate if the cases of exacerbation leading to ED or hospital admission were pure asthmatic re-accruals or were associated with infections, neutrophilia, or other signs of infection.

Altogether, our findings led us to speculate whether benralizumab might increase the risk of some infections in asthmatic patients due to its mechanism of action which targets IL-5 receptors instead of circulating IL-5 such as mepolizumab and reslizumab. While benralizumab inhibits the binding of IL-5 to its receptor and induces antibody-dependent cell cytotoxicity on IL-5R + cells [1], mepolizumab action could be exerted through reducing the mobilization of eosinophils from bone marrow, reducing the maturation of eosinophils from progenitors, having a different selectivity on different subset of eosinophils, and/or a return of eosinophils to peripheral blood from the tissues [37].

### 4.1 Study limitations

Our study presents some limitations and caveats. Events presented in RCTs are generally presented as “patient with event” over the total number of patients and are divided by different event categories. However, the same patient could be included in multiple, also similar, events. For this reason, we could not cumulate all respiratory tract infection events to avoid the possible artefactual duplication of included patients even when some events were similar in the classification (e.g., nasopharyngitis, pharyngitis, sinusitis, acute sinusitis). Also, as discussed before, we could not distinguish exacerbations associated with markers of eosinophilia (i.e., *anti*-IL-5 inefficacy) from exacerbations possibly associated to infections that may have elicited asthma worsening (i.e., a possible effect of *anti*-IL-5 in increasing the risk of some infections in a subset of individuals).

## 5 Conclusion

Our results suggest that *anti*-IL-5 treatments might have different effects on the reporting of some infection events in patients with asthma. The almost complete depletion of eosinophils by benralizumab might lead to an increased risk of some infections. However, the evidence is limited and these results are far from conclusive and strongly suggest the need of future studies to evaluate the risk of infections in patients with asthma receiving *anti*-IL-5 treatments.

## Data availability statement

The data supporting the findings of this study are available within the article. No further data were used for the realization of this study.

## CRediT authorship contribution statement

**Riccardo Giossi:** Writing – original draft, Methodology, Formal analysis, Data curation, Conceptualization. **Arianna Pani:** Writing – review & editing, Data curation, Conceptualization. **Jan Schroeder:** Writing – review & editing, Conceptualization. **Francesco Scaglione:** Writing – review & editing, Supervision, Conceptualization.

## Declaration of competing interest

The authors declare that they have no known competing financial interests or personal relationships that could have appeared to influence the work reported in this paper.
